# Atorvastatin Attenuates Programmed Death Ligand-1 (PD-L1) Induction in Human Hepatocellular Carcinoma Cells

**DOI:** 10.3390/ijms22168755

**Published:** 2021-08-15

**Authors:** Thuzar Hla Shwe, Peraphan Pothacharoen, Thanyaluck Phitak, Benjawan Wudtiwai, Prachya Kongtawelert

**Affiliations:** 1 Thailand Excellence Center for Tissue Engineering and Stem Cells, Department of Biochemistry, Faculty of Medicine, Chiang Mai University, Chiang Mai 50200, Thailand; thuzar.hs@gmail.com (T.H.S.); peraphan.pothacharoen@gmail.com (P.P.); thanyaluck.phitak@cmu.ac.th (T.P.); Benjawanwudtiwai@gmail.com (B.W.); 2 Department of Medical Research, Ministry of Health, Yangon P.O. Box 11191, Myanmar

**Keywords:** PD-L1, IFNγ, TNFα, atorvastatin, liver cancer, HepG2

## Abstract

Liver cancer is the sixth most common cancer worldwide with high morbidity and mortality. Programmed death ligand 1 (PD-L1) is a major ligand of programmed death 1 receptor (PD1), and PD1/PD-L1 checkpoint acts as a negative regulator of the immune system. Cancers evade the host’s immune defense via PD-L1 expression. This study aimed to investigate the effects of tumor-related cytokines, interferon gamma (IFNγ), and tumor necrosis factor alpha (TNFα) on PD-L1 expression in human hepatocellular carcinoma cells, HepG2. Furthermore, as atorvastatin, a cholesterol-lowering agent, is documented for its immunomodulatory properties, its effect on PD-L1 expression was investigated. In this study, through real-time RT-PCR, Western blot, and immunocytochemistry methods, PD-L1 expression in both mRNA and protein levels was found to be synergistically upregulated in HepG2 by a combination of IFNγ and TNFα, and STAT1 activation was mainly responsible for that synergistic effect. Next, atorvastatin can inhibit the induction of PD-L1 by either IFNγ alone or IFNγ/TNFα combination treatment in HepG2 cells. In conclusion, in HepG2 cells, expression of PD-L1 was augmented by cytokines in the tumor microenvironment, and the effect of atorvastatin on tumor immune response through inhibition of PD-L1 induction should be taken into consideration in cancer patients who have been prescribed atorvastatin.

## 1. Introduction

Being the sixth most commonly diagnosed cancer and the third leading cause of cancer death in 2020, liver cancer has been imposing a significant burden on world health [1]. Moreover, hepatocellular carcinoma (HCC), the major form of primary liver cancer, is known for its progressiveness and poor prognosis. The majority of HCC patients are diagnosed at advanced stages when most therapeutic options such as transplant, surgery, or ablation are impractical [2]. Finding out newer treatment regimens has become a necessity, and immunotherapy is one of them.

Progression of hepatocellular carcinoma is a multistep process in which inflammation plays a critical component, and many cytokines and chemokines take part in linking inflammation and cancer [3]. Inflammation-prone tumor microenvironment (TME) of HCC is associated with conditions favoring tumor immune tolerance such as the high ratio of T regulatory cells, secretion of immunoregulatory cytokines, or expression of immune inhibitory molecules [2,4]. Understanding these influencing factors is of value for the therapeutic strategies of HCC.

Tumor immune tolerance is a phenomenon by which a tumor escapes from the scrutiny of the host immune system. Programmed death ligand 1 (PD-L1), also known as B7-H1 or CD274, is the major ligand of programmed death receptor 1 (PD1), one of the immunosuppressive co-inhibitory receptors on T cells (immune checkpoint). Engagement between PD-L1 and PD1 strongly counteracts T-cell receptor (TCR) signal transduction and CD80/CD28 co-stimulation, resulting in weaning off the effector responses of the immune system [5]. PD-L1 is normally expressed at a low level in immune cells and immune-privileged tissues and is induced during inflammation or infection in a variety of the cells as a counter-regulatory suppressive signal [6]. Many tumors exploit this negative immune regulatory mechanism by upregulating the expression of PD-L1, favoring their survival. In many epithelial tumors, including HCC, PD-L1expression is reported to be increased; hence, PD-L1 becomes a popular target for tumor immunotherapy [7]. PD-L1 can either be constitutively expressed over genetic alteration or induced by external factors in TME. In the case of HCC, PD-L1 expression is mainly attributed to the latter mechanism. Extrinsic induction of PD-L1 in tumors is generally mediated by proinflammatory cytokines derived from tumor-infiltrating lymphocytes (TILs), mainly interferon gamma (IFNγ). Studies in diverse cell types exhibited potent induction of PD-L1 by IFNγ [8,9,10]. Likewise, tumor necrosis factor-α (TNFα), a proinflammatory cytokine related to hepatic inflammation, can increase PD-L1 levels [11,12]. Moreover, TNFα was reported to synergize with IFNγ in the induction of PD-L1 in human HCC cells, SMMC-7721, BEL-7402, HuH-7, and Hep-3B [13]. These studies stated that many pathways such as JAK/STAT and NFкB pathways are involved in the induction of PD-L1; therefore, inhibiting these pathways involved might be therapeutically of value in HCC. However, there was no study about the effect of combined inhibition of these pathways on PD-L1 expression. In this study, we confirmed that TNFα significantly augments the PD-L1-inductive effect of IFNγ in HepG2 cells, a human hepatocellular carcinoma cell line, and this effect was through activation of STAT1, JNK, and NFкB pathways. Moreover, inhibition of STAT1 activation alone was enough as combined inhibitions with other responsible pathways showed no further significant inhibitory effect on PD-L1 expression.

Atorvastatin is a commonly used lipid-lowering agent for its function in the mevalonate pathway of cholesterol synthesis as an inhibitor of the rate-limiting enzyme 3-hydroxy-3-methylglutaryl coenzyme A reductase (HMG-CoA reductase) [14]. In addition to their original usage to lower the cardiovascular risk, statin use is also related to the reduction in cancer risk, including HCC proven by several meta-analyses [15], while the immunomodulatory effect of statins is suggested to be one of the reasons for its anticancer effect [16,17]. As atorvastatin was demonstrated to dose-dependently inhibit STAT1 phosphorylation [18], we hypothesized that atorvastatin might have an effect on the IFNγ induced expression of PD-L1 in HCC cells. In this study, we found that atorvastatin lowers the expression of PD-L1 in IFNγ-induced HepG2 cells significantly and dose-dependently.

## 2. Results

### 2.1. PD-L1 Expression Is Synergistically Upregulated by Combination of IFNγ and TNFα in HepG2 Cells

**Figure 1:**
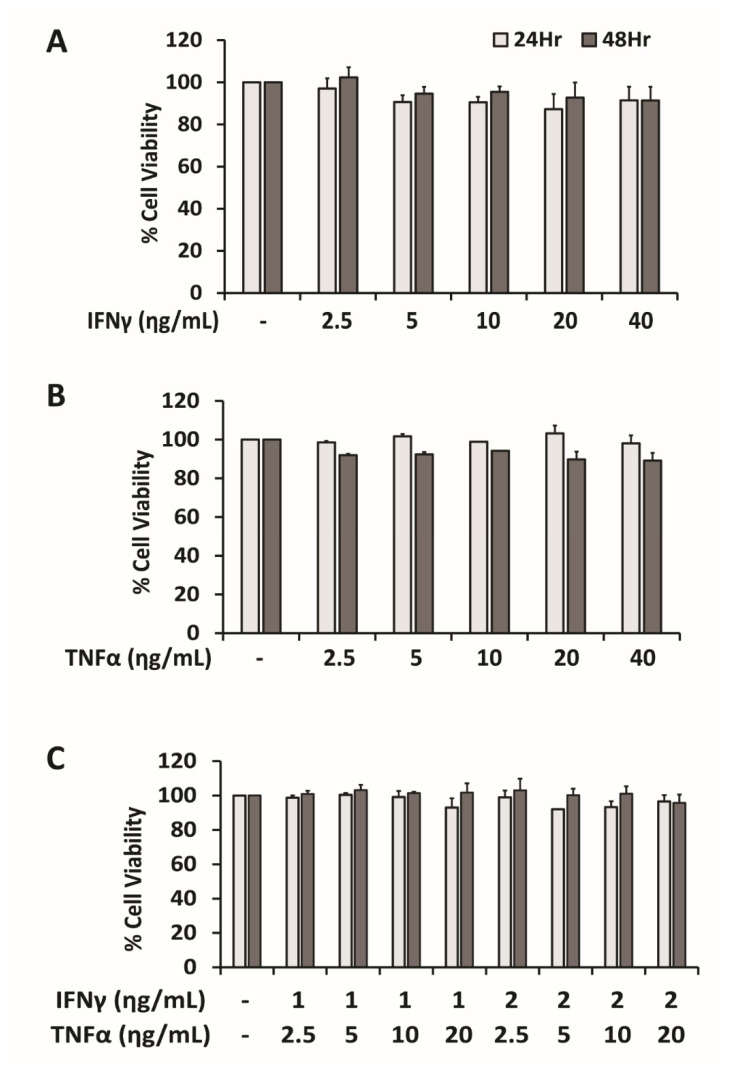
Individual or combination treatments of cytokines: IFNγ and/or TNFα are not toxic to HepG2 cells. HepG2 cells were treated with either IFNγ (**A**) or TNFα (**B**), or a combination of IFNγ and TNFα (**C**) at various concentrations (ng/mL) for 24 and 48 h and percentage of cell viability was assessed by MTT assays. Data are presented as mean ± SD, *n* = 3.

The difference between the effect of individual cytokine (IFNγ or TNFα) and that of a combination of these two cytokines on programmed death ligand-1 (PD-L1) expression in HepG2 cells was determined in this study. Firstly, MTT assay was performed at 24- and 48-h period of treatment to find out the non-toxic dose of IFNγ and TNFα: either individual or combination treatment, in HepG2 cells (Figure 1A–C). Either IFNγ or TNFα up to 40 ng/mL concentration was not toxic to HepG2 cells in both 24 and 48 h incubation periods based on the results of more than 80% cell viability. Combination treatment of IFNγ 2 ng/mL and TNFα 20 ng/mL concentration for 24 and 48 h treatment duration was also proved to be tolerable for further experiments.

**Figure 2:**
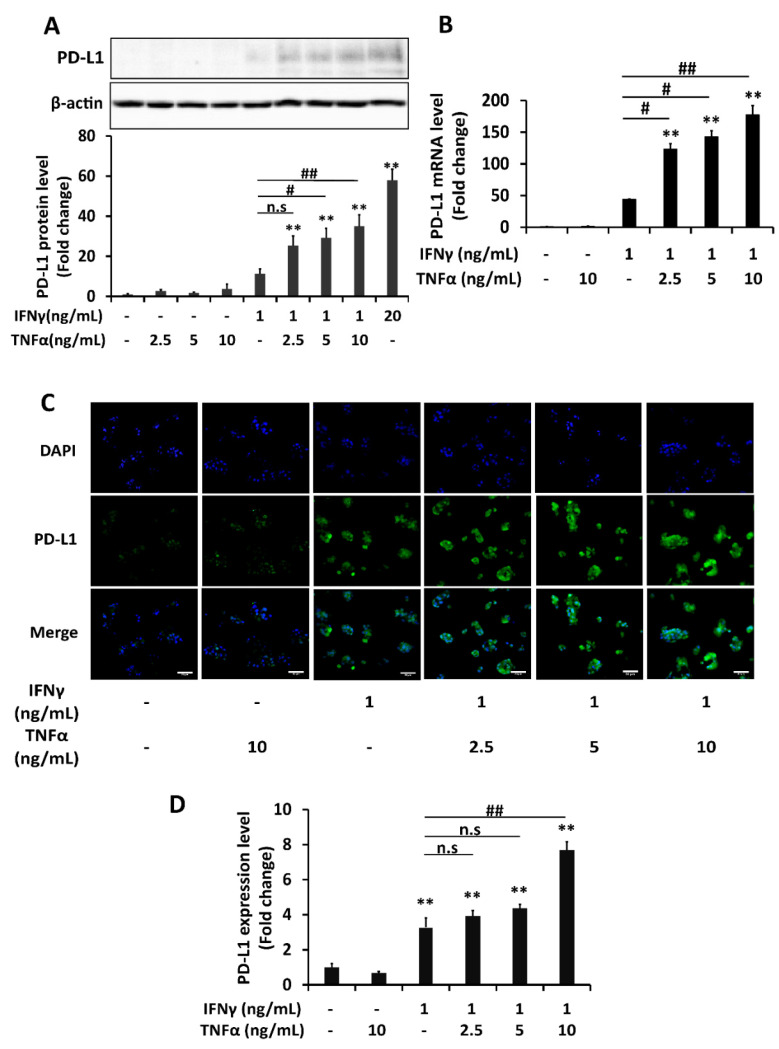
Combination treatment of IFNγ and TNFα synergistically upregulates PD-L1 expression both in mRNA and protein level in HepG2 cells. (**A**–**D**) Analysis of PD-L1 expression of HepG2 cells treated with either individual or combination of IFNγ and TNFα at various concentrations (ng/mL) for 6 h in RT-PCR analysis and for 24 h in Western blot or immunocytochemistry analysis. (**A**) Western blot analysis and quantification. β-actin was used as a loading control. (**B**) RT-PCR analysis. Relative PD-L1 mRNA level was normalized to the expression of the GAPDH gene. (**C**,**D**) Immunocytochemistry of PD-L1 membrane expression and its quantification. Scale bar = 50 μm. Data are presented as mean ±SD for WB and RT-PCR; mean ±SEM for Immunocytochemistry. *n* = 3. * *p* < 0.05, ** *p* < 0.01 compared to control. # *p* < 0.05, ## *p* < 0.01 compared to IFNγ single treatment.

In the next experiment, HepG2 cells were treated with either individual or combination of IFNγ and TNFα to determine the alterations in PD-L1 expression. The western blot results showed that IFNγ 1 ng/mL upregulated PD-L1 protein expression. Although TNFα treatment (2.5–10 ng/mL) did not significantly affect PD-L1 protein expression itself, it significantly augmented the effect of IFNγ on PD-L1 in a dose-dependent manner. The addition of 2.5 ng/mL of TNFα induced PD-L1 levels more than two times as much as induced by single IFNγ treatment. IFNγ 20 ng/mL that was known to be able to induce PD-L1 expression was used as a positive control (Figure 2A). These findings are consistent with the induction of mRNA level of PD-L1 by a combination of these cytokines in real-time PCR analysis (Figure 2B). The membrane PD-L1 level in the immunocytochemistry experiment also confirmed a similar pattern of induction, although the extent of augmentation was not that significant in 2.5 and 5 ng/mL concentrations of TNFα (Figure 2C,D). These findings suggested that a combination of IFNγ and TNFα cytokines synergistically upregulate the mRNA and protein expression of PD-L1 in HepG2 cells significantly.

### 2.2. Activation of JNK of MAPK Pathway and NFĸB in Addition to STAT1 Is Responsible for Augmented Upregulation of PD-L1 by Combination Treatment of IFNγ and TNFα

**Figure 3:**
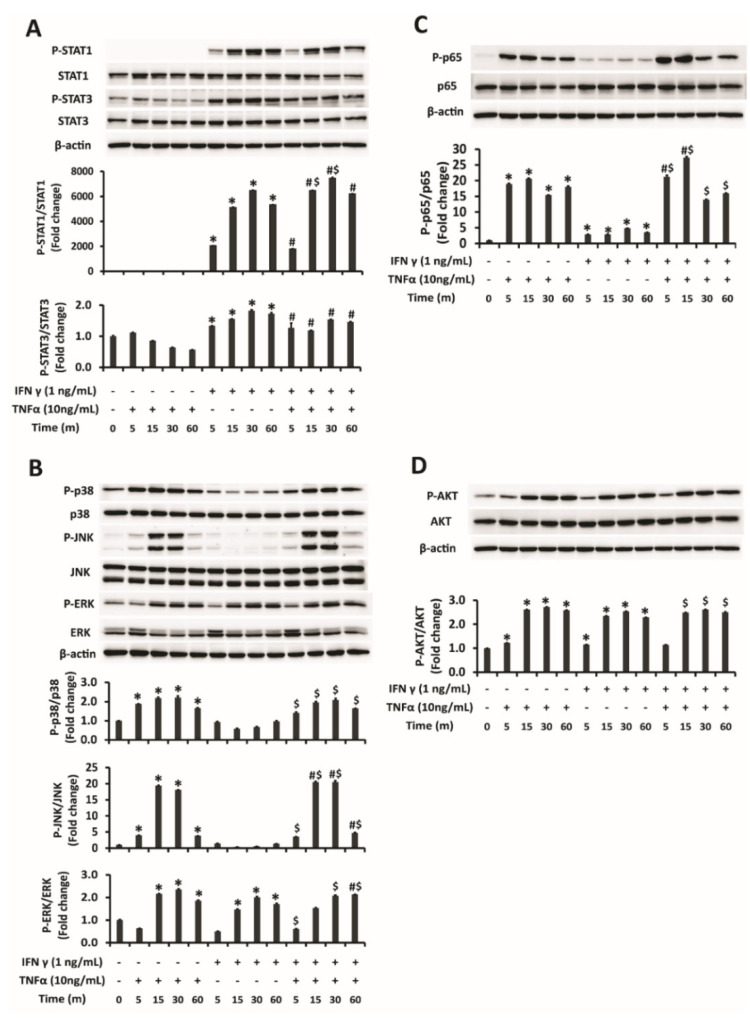
Activation of signaling pathways by combination treatment of IFNγ and TNFα. (**A**–**D**) Western blot analysis and quantification of the phosphorylation status of signaling proteins in HepG2 cells induced by combination treatment of IFNγ 1 ng/mL and TNFα 10 ng/mL for 5, 15,30 and 60 min. (**A**) STAT1 and STAT3 (**B**) MAPK pathway: JNK, p38, and ERK (**C**) NFκB, and (**D**) Akt activation. The density ratio of proteins was shown as relative expression of phosphorylated form to respective total form. β-actin was used as a loading control. Data are presented as mean ± SD. *n* = 3. * *p* < 0.05 compared to control. # *p* < 0.05 compared to TNFα treatment of respective time points. $ *p* < 0.05 compared to IFNγ treatment of respective time point.

Signaling pathways responsible for the synergistic PD-L1 upregulation by IFNγ and TNFα combination were determined in this study. While IFNγ single treatment most significantly induced STAT1 phosphorylation in addition to activation of STAT3, ERK of MAPK pathway and Akt, TNFα single treatment showed almost no effect on STAT1 activation but activated inflammatory proteins: JNK, p38 and ERK of MAPK pathway, NFκB and Akt phosphorylation (Figure 3A–D). As for the IFNγ and TNFα combination treatment, it triggered significantly higher phosphorylation of STAT1, JNK, and NFκB pathways comparing with either IFNγ or TNFα single treatment of respective time point (Figure 3A–C). It showed that enhanced activation of STAT1, JNK, and NFκB pathways may be accountable for the amplified upregulation of PD-L1 expression by IFNγ and TNFα combination compared to single treatments of either cytokine.

### 2.3. STAT1 Signaling Is Mainly Responsible for Enhanced Upregulation of PD-L1 by Combination Treatment of IFNγ and TNFα

**Figure 4:**
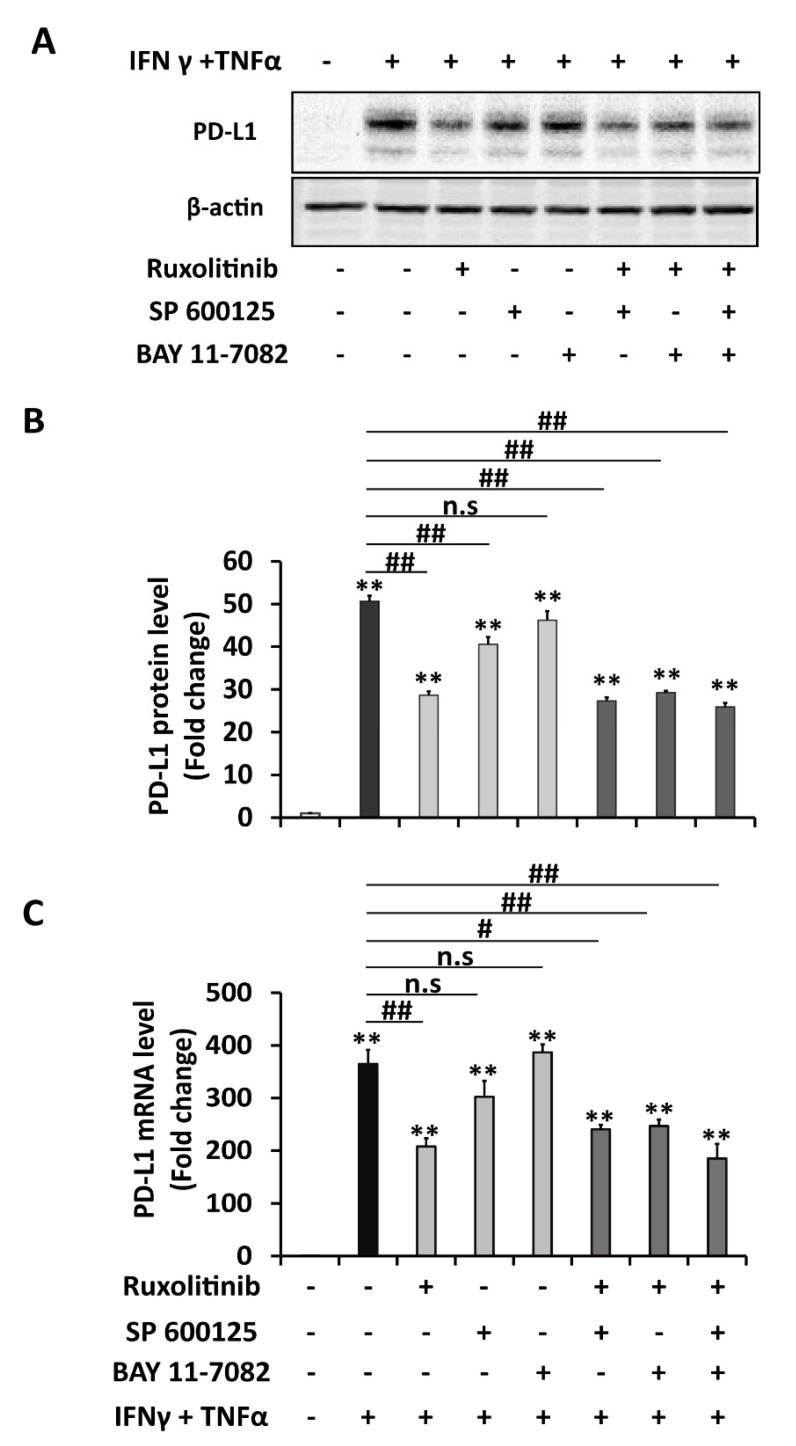
Enhanced upregulation of PD-L1 expression by combination treatment of IFNγ and TNFα compared to individual cytokine treatment is mainly dependent on STAT-1 signaling pathway. (**A**–**C**) Analysis of PD-L1 expression of HepG2 cells pre-treated for 2 h with individual or combination of inhibitors: JAK inhibitor (ruxolitinib 1 μM), JNK inhibitor (SP 600125 10 μM), NF-κB inhibitor (BAY 11-7082), which was followed by treatment with a combination of IFNγ 1 ng/mL and TNFα 10 ng/mL for 6 h in RT-PCR analysis, and for 24 h in Western blot analysis. (**A**,**B**) Western blot analysis and quantification. β-actin was used as a loading control. (**C**) RT-PCR analysis. Relative PD-L1 mRNA level was normalized to the expression of the GAPDH gene. Data are presented as mean ± SD. *n* = 3. * *p* < 0.05, ** *p* < 0.01 compared to control. # *p* < 0.05, ## *p* < 0.01 compared to IFNγ and TNFα combined treatment.

**Figure 5:**
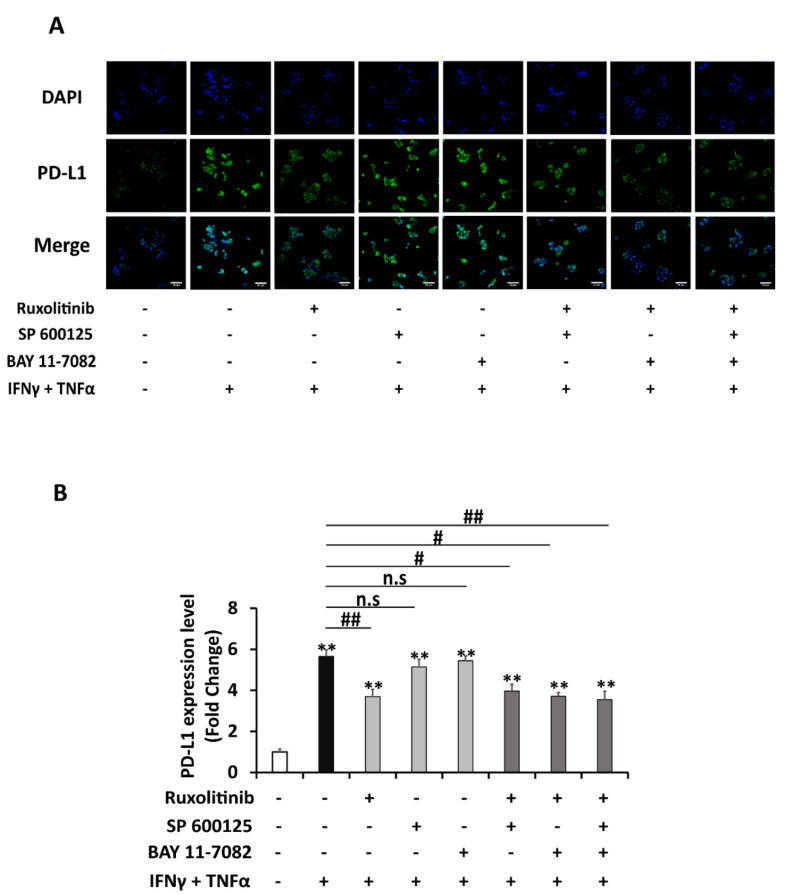
STAT-1 signaling is the major pathway for the upregulation of PD-L1 expression by combination treatment of IFNγ and TNFα. (**A**) Representative figure and (**B**) quantification of immunocytochemistry analysis of PD-L1 membrane expression in HepG2 cells pre-treated for 2 h with individual or combination of inhibitors: JAK inhibitor (ruxolitinib 1 μM), JNK inhibitor (SP 600125 10 μM), NF-κB inhibitor (BAY 11-7082), which was followed by treatment with a combination of IFNγ 1 ng/mL and TNFα 10 ng/mL for 24 h. Scale bar = 50 μm. Data are presented as mean ± SEM. *n* = 3. * *p* < 0.05, ** *p* < 0.01 compared to control. # *p* < 0.05, ## *p* < 0.01 compared to IFNγ and TNFα combined treatment.

In this experiment, commercial inhibitors of three pathways found to be enhanced by a combination of IFNγ and TNFα in the previous experiment: STAT1 inhibitor (ruxolitinib), JNK inhibitor (SP 600125), and NFκB inhibitor (BAY 11-7082) were used to verify the main responsible pathway(s) for the synergistic effect between IFNγ and TNFα. As shown in Figure 4A,B, pre-treatment with 1 μM concentration of ruxolitinib significantly reduced almost half (43%) of the combination treatment-induced PD-L1 expression level, whereas SP 600125 (10 μM) lowered 20% of it. In contrast, the NFκB inhibitor, BAY 11-7082 (10 μM), did not show a significant reduction in PD-L1 levels. Next, the effect of combined inhibition of STAT1, JNK, and NFκB pathways on PD-L1 expression was also determined. Although combined-inhibitors treatments, either two or three inhibitors, can reduce substantially of induced PD-L1 levels, there was no significant further reduction compared to the inhibition elicited by the single STAT1 inhibitor. This result implied that STAT1 inhibitor alone is effective in inhibiting PD-L1 expression. A similar pattern of findings on inhibition of PD-L1 expression was established in mRNA level (Figure 4C) and in membrane PD-L1 level (Figure 5A,B). These results revealed that enhanced upregulation of PD-L1 expression by combination treatment of IFNγ and TNFα compared to individual treatments is mainly acting through a STAT-1 signaling pathway.

### 2.4. Atorvastatin Inhibits Induction of PD-L1 Expression in HepG2 Cells

**Figure 6:**
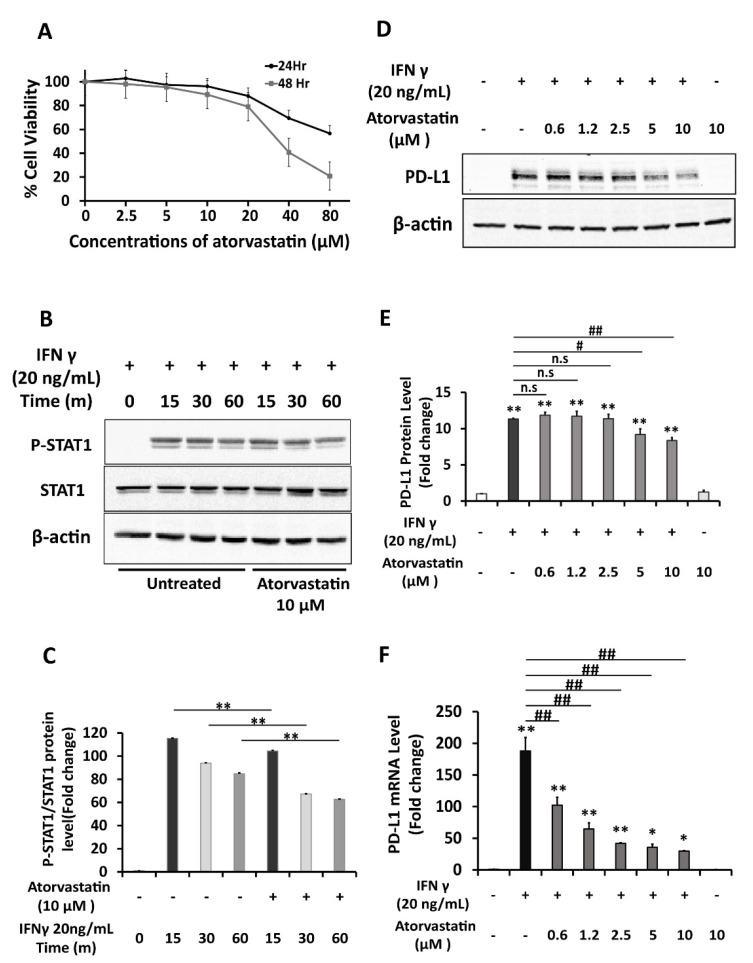
Induction of PDL1 expression by IFNγ is diminished by Atorvastatin, STAT-1 inhibitor, in HepG2 cells. (**A**) MTT assay for the viability of HepG2 cells treated with various concentrations of atorvastatin (μM) for 24 or 48 h. (**B**,**C**) Analysis of the effect of atorvastatin on phosphorylation of STAT-1 protein in HepG2 cells pre-treated with atorvastain 10 μM for 4 h, followed by induction with IFNγ 20 ng/mL for 15, 30, 60 min by western blot. The density ratio of protein was shown as relative expression of phosphorylated form to the total form of STAT1. β-actin was used as a loading control. (**D**–**F**) Analysis of PD-L1 expression of HepG2 cells co-treated with IFNγ 20 ng/mL and atorvastatin at various concentrations (μM) for 6 h in RT-PCR analysis, and for 24 h in western blot analysis. (**D,E**) Western blot analysis and quantification. β-actin was used as a loading control. (**F**) RT-PCR analysis. Relative PD-L1 mRNA level was normalized to the expression of the GAPDH gene. Data are presented as mean ± SD. *n* = 3. * *p* < 0.05, ** *p* < 0.01 compared to control. # *p* < 0.05, ## *p* < 0.01 compared to IFNγ treatment.

**Figure 7:**
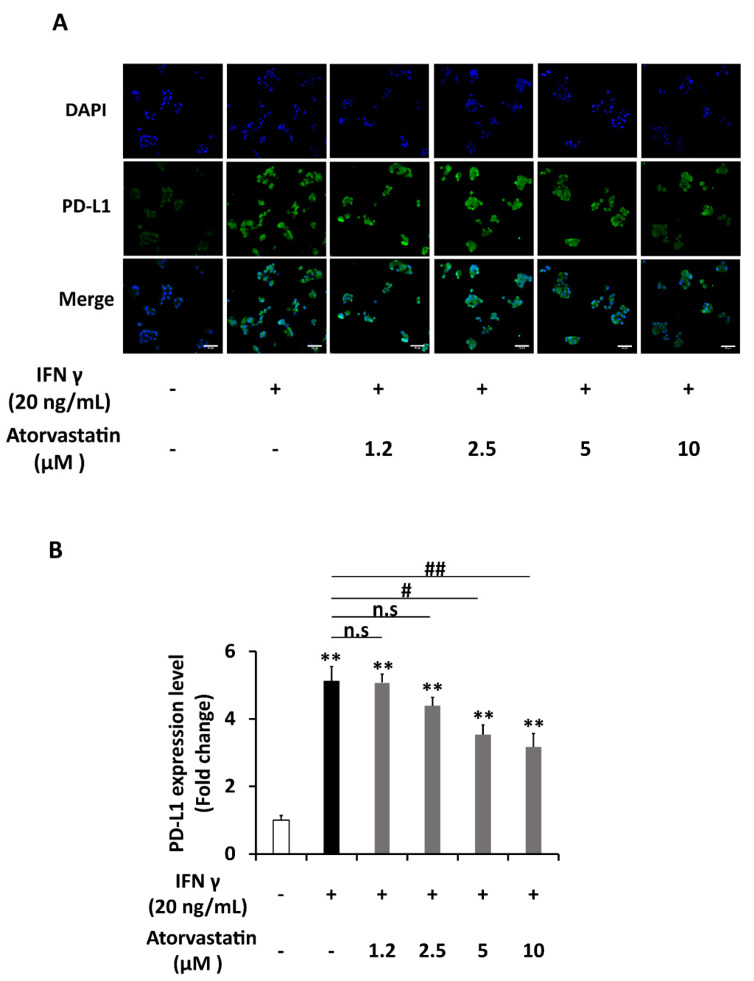
Atorvastatin mitigate the effect of IFNγ on upregulation of PD-L1 expression in HepG2 cells. (**A**) Representative figure and (**B**) quantification of immunocytochemistry analysis of PD-L1 membrane expression in HepG2 cells co-treated with IFNγ 20 ng/mL and atorvastatin at various concentrations (μM) for 24 h. Scale bar = 50 μm. Data are presented as mean ± SEM. *n* = 3. * *p* < 0.05, ** *p* < 0.01 compared to control. # *p* < 0.05, ## *p* < 0.01 compared to IFNγ treatment.

**Figure 8:**
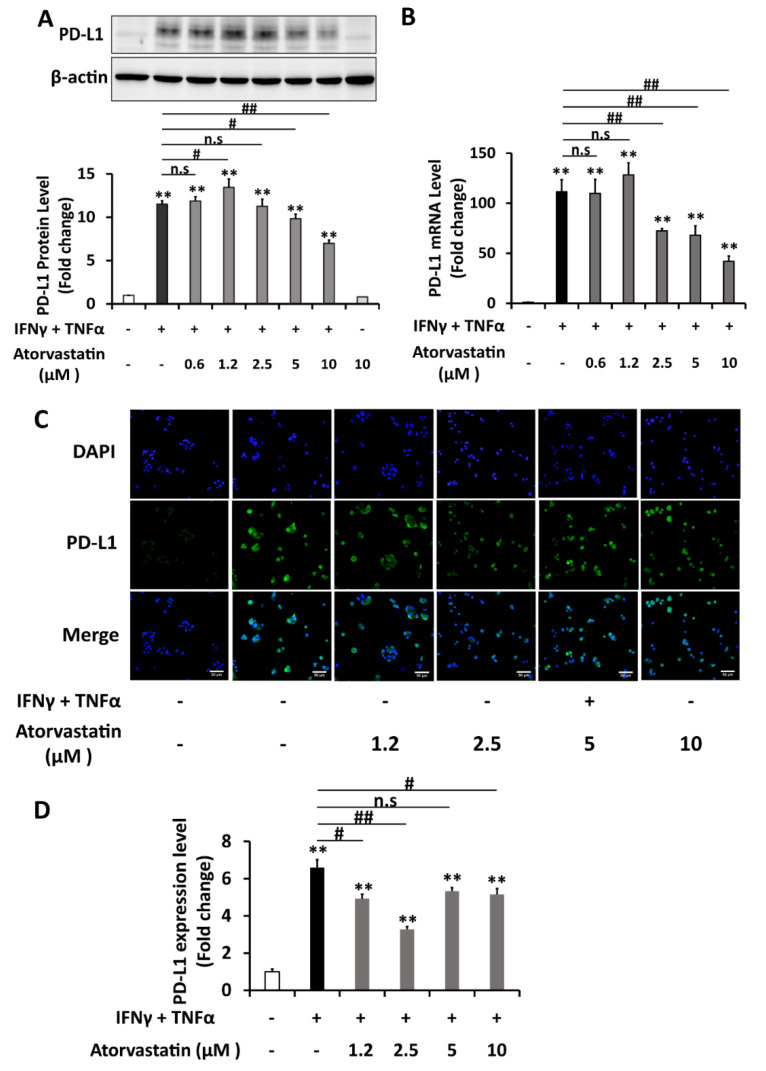
Atorvastatin reduces the induction of PD-L1 expression by IFNγ and TNFα combined treatment in HepG2 cells. (**A**–**D**) Analysis of PD-L1 expression of HepG2 cells co-treated with a combination of IFNγ 1 ng/mL+TNFα 10 ng/mL and atorvastatin at various concentrations (μM) for 6 h in RT-PCR analysis, and for 24 h in western blot and immunocytochemistry analysis. (A) Western blot analysis and quantification. β-actin was used as a loading control. (**B**) RT-PCR analysis. Relative PD-L1 mRNA level was normalized to the expression of the GAPDH gene. (**C**,**D**) Immunocytochemistry of PD-L1 membrane expression and its quantification. Scale bar = 50 μm. Data are presented as mean±SD for WB and RT-PCR; mean±SEM for immunocytochemistry. *n* = 3. * *p* < 0.05, ** *p* < 0.01 compared to control. # *p* < 0.05, ## *p* < 0.01 compared to IFNγ and TNFα combined treatment.

Previous studies stated that atorvastatin can inhibit the phosphorylation of STAT1 under the induction of IFNγ [18,19]. As STAT1 signaling is crucial in PD-L1 upregulation, the effect of atorvastatin on PD-L1 expression was explored in HepG2 cells. MTT assays showed that atorvastatin concentrations up to 10 μM were not toxic to HepG2 cells in both 24- and 48-hours incubation periods (Figure 6A). Atorvastatin was firstly proved to significantly inhibit the IFNγ-induced phosphorylation of STAT1 in HepG2 cells (Figure 6B,C). Next, HepG2 cells were treated with various concentrations of atorvastatin either in the presence of IFNγ 20ng/mL or a combination of IFNγ 1 ng/mL and TNFα 10 ng/mL. The results showed that atorvastatin of 5 and 10 μM concentrations significantly reduced the PD-L1 protein expression induced by both types of co-treatment. However, the reduction in PD-L1 protein was not seen in low doses of atorvastatin when the cells were induced by combined IFNγ and TNFα treatment (Figure 6D,E and Figure 8A). As for the membrane PD-L1 levels determined by the immunocytochemistry method, atorvastatin can inhibit when it was induced by IFNγ individual treatment significantly and dose-dependently. However, this inhibitory effect was not dose-dependent when cells were induced by combination treatment (Figure 7A,B and Figure 8C,D). The ability of atorvastatin to inhibit PD-L1 induction was more obvious in mRNA level since almost half of the level of PD-L1 mRNA induced by IFNγ treatment was lowered by atorvastatin concentration as low as 0.6125 μM (Figure 6F). Once again, low atorvastatin doses cannot inhibit the mRNA level of PD-L1 induced by combination treatment as well as it can inhibit when the cells were induced by IFNγ single treatment (Figure 8B). From these, it was demonstrated that atorvastatin impedes STAT1 activation, thereby reducing the expression of PD-L1 in both transcription and translation levels in hepatocellular carcinoma cells.

## 3. Discussion

There is heightened interest in adaptive immune resistance contributed by PD-L1 induction in cancer, the mechanism by which cancer cells evade themselves from T cell-mediated destruction of the host [20]. Studies have shown that local inflammatory stimuli, such as cytokines released by infiltrating immune cells in the TME, can influence PD-L1 expression, where IFNγ is claimed to be the most potent one. Understanding mechanisms of PD-L1 induction inflicted by factors in the TME suggest new strategies to mitigate PD-L1 expression in TME, favoring improvement of cancer therapy [21]. While constitutive expression of PD-L1 in HepG2 cells is controversial [22,23,24], this study confirmed that PD-L1 expression is only inducible in HepG2; PD-L1 can be detected only after induction with IFNγ. While many studies stated that IFNγ can upregulate PD-L1 expression significantly, the pathways responsible for the upregulation depend on cell type. IFNγ from the type II interferon family is mainly secreted by activated T cells, NK cells, and macrophages in TME. IFNγ binds to its receptor, the interferon gamma receptor (IFNGR), which leads to JAK1 and JAK2 activation and subsequent recruitment and activation of transcription factors STAT1 and STAT3 [25]. IFNγ can also elicit other pathways, including MAPkinase, NFĸB, or PI3-Akt pathways in a gene- and cell type-specific manner [26]. In gastric cancer, head and neck cancers, and cholangiocytes, IFNγ induces PD-L1 expression through the JAK/STAT1 pathway [27,28,29]. However, JAK/STAT3 and PI3K-AKT signaling pathways are responsible for IFNγ-induced PD-L1 induction and immune escape in lung cancer [30]. In dermal fibroblast, PD-L1 expression is induced by IFNγ through transient activation of MAPK and PI3K pathways, which in turn induces NFĸB translocation to PD-L1 promoter [31]. However, IFNγ-induced PD-L1 expression in multiple myeloma cells is mainly through MEK/ERK pathway, while the JAK-STAT pathway shows only a weak influence and PI3K/AKT and NFĸB pathways show no influence at all [32]. In our study, PD-L1 expression was inducible through IFNγ-mediated activation of STAT1 mainly. Although it can also activate STAT3, MEK/ERK, and PI3K/AKT pathways, the effect was trivial.

As chronic inflammation plays an important role in hepatocellular carcinogenesis, the proinflammatory cytokine TNFα, which is mainly secreted by macrophages, is involved in cancer cell proliferation [3]. Additionally, recent studies have shown that TNFα also induces the expression of PD-L1 to promote tumor escape. TNFα increases PD-L1 expression in murine tumor-associated monocytes [12] and can also improve PD-L1 stability via the NFĸB pathway in cancer cells [11]. However, in our study, TNFα did not upregulate PD-L1 significantly because of the low dose we used probably. Additionally, TNFα can also augment the effect of IFNγ, the principal cytokine of PD-L1 induction. That synergism is reported to be through activation of the NFкB pathway in myelodysplastic blasts [33]. In human HCC cells SMMC-7721, BEL-7402, HuH-7, and Hep-3B, TNFα synergizes with IFNγ in inducing PD-L1 through the induction of IFNγ receptors expression via NFкB pathway, thereby enhancing JAK/STAT signaling of IFNγ [13]. Our study in another HCC cell line, HepG2, confirmed that TNFα and IFNγ synergistically act together on the expression of PD-L1, and the main mechanism is through phosphorylation of STAT1 in addition to JNK and NFкB in accordance with the previous studies. In this study, we explored the effect of combined inhibition of involving pathways as well to improve the understanding of the role of signaling pathways that can, in turn, be useful in developing therapeutic strategies. Here, combined inhibition of STAT1 and JNK and/or NFкB showed no better inhibition than that of STAT1 signaling alone, implying STAT1 inhibitors would be enough to impede PD-L1 expression. From these results, we can say that tumor escape from the immune system is further facilitated by inflammatory cytokines, which are abundant in the TME of inflammation-prone tumors such as HCC, and it can be countered by using STAT1 inhibitors.

Atorvastatin is a competitive inhibitor of the key enzyme of cholesterol synthesis, HMG-CoA reductase. Mevalonate, the immediate product of HMG-CoA reductase, is phosphorylated by mevalonate kinase to form isopentenyl pyrophosphate (IPP), which is further converted to isoprenoids such as farnesyl pyrophosphate (FPP) and geranylgeranyl pyrophosphate (GGPP). Then, FPP is converted to squalene and then to cholesterol in the cascade. The isoprenoids intermediates are important for post-translational modification of GTP-binding proteins such as Rho and Ras, which are important for cell cytoskeletal structure, signaling, cell motility, protein trafficking, and membrane transport [34]. Atorvastatin, which is a commonly used cholesterol-lowering drug, is related to the reduction in cancer risk, including HCC, according to several meta-analyses [15]. Moreover, concomitant prescription of statins presented an increased response rate to an anti-PD-L1 checkpoint inhibitor, nivolumab, in non-small cell lung cancer patients [35]. Atorvastatin was demonstrated to inhibit phosphorylation of STAT1 imposed by IFNγ in mononuclear cells of multiple myeloma patients; hence, we were convinced to find its effect on PD-L1 expression in HepG2 cells in this study [18]. A study in peripheral blood mononuclear cells stated that statins can downregulate the expression of co-inhibitory receptors on T cells such as PD-1, CTLA-4, TIM-3, and LAG-3 and that of the ligand, PD-L1 when the cells were co-treated with atorvastatin and lipopolysaccharide (LPS) at 48 and 72 h [36]. However, the effect of atorvastatin treatment on PD-L1 expression in cancer cells has yet to be reported. On top of the confirmation of the inhibition of STAT1 activation induced by IFNγ, our study firstly stated that atorvastatin diminishes IFNγ-induced PD-L1 expression in both mRNA and protein levels significantly and dose-dependently in HepG2 cells. Atorvastatin alone had shown no effect on PD-L1 levels. We also investigated the effect of atorvastatin on the levels of PD-L1 induced by combination treatment of IFNγ and TNFα, and it was inhibitory as well in both translation and transcription levels. However, the inhibitory effect was not dose-dependent in the case of combined induction of two cytokines, especially in low doses. It was probably due to the differential effect of atorvastatin on phosphorylation of STAT1 at different sites. Atorvastatin can block STAT1 phosphorylation at the tyrosine site (Y-701) but not that at the serine site (S-727), while the serine site was mainly phosphorylated by p38, JNK, ERK1/2 of MAPK pathway, and the mROR pathway [18,19]. TNFα treatment was shown to activate these three MAPKinases, and among them, JNK was significantly enhanced by combination treatment in this study. Therefore, low doses of atorvastatin may not effectively inhibit the tyrosine phosphorylation of STAT1, whereas STAT1 is activated by phosphorylation at both sites carrying out its full function.

Concerning the relationship between STAT-1 activation and atorvastatin, Lee et al. demonstrated that simvastatin, another type of statin, can inhibit STAT-1 activation through inhibition of the mevalonate pathway, but that inhibitory effect is independent of cholesterol. Mevalonate, the immediate product of HMG-CoA reductase, and its downstream isoprenoids, geranylgeranyl pyrophosphate (GGPP), can rescue the inhibition of STAT1 activation by simvastatin while cholesterol, the final product, has no effect [37]. Therefore, inhibition of STAT1 by atorvastatin may also be through inhibition of mevalonate pathway intermediates.

On the other hand, inhibition of cholesterol synthesis can downregulate the expression of PD-1 co-inhibitory receptors in T cells through reduction in ER stress, thereby improving T-cell exhaustion [38]. Hence, reduction in cholesterol content might also be responsible for the reduction in PD-L1 expression by atorvastatin in cancer cells, influencing the immune response of the T cells.

PD-L1 inhibitory effect of atorvastatin might benefit the cancer treatment since drugs inhibiting eIF4F–STAT1–PD-L1 axis mediates tumor regression in the murine melanoma model [39]. In contrast, some studies stated that absent activity of or insensitivity to IFNγ pathway can lead to lack of IFNγ-induced surface expression of MHC class I in addition to PD-L1, resulting in disruption of antigen-presenting machinery to T cells. This phenomenon might attenuate the capacity of anti-PD1/PD-L1 blockade on immune rescue [40]. Therefore, the effect of atorvastatin on STAT1 inhibition, which in turn inhibits PD-L1 expression, should be further investigated to validate its effect on the treatment response if they were prescribed together.

Our study indubitably has some limitations. Firstly, although TNFα can stabilize PD-L1 protein by inhibiting the breakdown via ubiquitination in cancer cells [11], we did not tackle the effect of cytokines on the stability and membrane translocation of PD-L1. It might contribute to the different inhibitory patterns of atorvastatin on membrane PD-L1 levels of IFNγ induction and that of combination treatment. Secondly, the effect of atorvastatin on the signaling pathways other than STAT1 in HepG2 cell type has not been explored. Moreover, there was no investigation about the actual effect of atorvastatin on activation of immune cells, T cells, neither in vivo nor in vitro. In future research, the signaling mechanisms accountable for the effect of atorvastatin should be explored, and the effect of atorvastatin on T cells activity should be investigated.

In summary, the inflammatory cytokine in the tumor microenvironment TNFα can synergistically augment the effect of T cells-derived cytokine, IFNγ, on the expression of immunosuppressive ligand, PD-L1 in HepG2 cells favoring tumor immune escape phenomenon. The synergy between two cytokines was mainly through the JAK/STAT1 pathway, and inhibition of this pathway alone substantially deters the expression of PD-L1. Furthermore, the commonly prescribed lipid-lowering atorvastatin had shown to diminish the PD-L1 induction by IFNγ alone or IFNγ/TNFα combination, implying that the use of atorvastatin might be helpful in developing therapeutic strategies to counter the immune evasion in cancers.

## 4. Materials and Methods

### 4.1. Reagents

Dulbecco’s Modified Eagle’s Medium—high glucose (DMEM-HG), fetal bovine serum (FBS), and trypsin-EDTA solution were purchased from Gibco (Grand Island, NY, USA). Recombinant human IFNγ and TNFα were obtained from Pepro Tech (Rocky Hill, NJ, USA). Atorvastain calcium (PHR1422), 3-(4,5-dimethythiazol-2-yl)-2,5-diphenyltetrazolium bromide bromide (MTT), and dimethyl sulfoxide (DMSO) were purchased from Sigma Chemical, Inc. (St Louis, MO, USA). Primary antibodies against PD-L1 (13684), signaling proteins (phosphorylated and total forms of STAT1 (9167)(9175), p65 (3033)(8242), Akt (9271)(4691), ERK (9101)(9102), p38 (9211)(9212) and JNK (9251)(9252)), actin (4970) and anti-rabbit immunoglobulin G (IgG)-conjugated horse-radish peroxidase (HRP) (7074) were purchased from Cell Signaling Technology, Inc. (Beverly, MA, USA). Signaling inhibitors: Jak inhibitor (Ruxolitinib: 83405), JNK inhibitor (SP 600125: 8177), and NFκB inhibitor (Bay 11-7082: 78679) were also obtained from Cell Signaling Technology, Inc. (Beverly, MA, USA). Goat anti-rabbit IgG-FITC (Alexa Fluor 488: ab150077) and phosphorylated and total forms of STAT3 (ab76315) (ab119352) were purchased from Abcam (San Francisco, CA, USA).

### 4.2. Cell Culture

The human hepatoblastoma cell line HepG2 was purchased from American Type Culture Collection (ATCC) (Manassas, VA, USA). Cells were cultured in high glucose DMEM media supplemented with 10% fetal bovine serum, penicillin/streptomycin (Gibco, Grand Island, NY, USA), and gentamycin at 37 °C of a humid atmosphere incubator with 5% CO_2_. Cells were harvested for the treatments or further passaging when they reached 70–80% confluence.

### 4.3. Cytotoxic Assay

MTT assay was used to determine cellular viability against the treatments of IFNγ and/or TNFα and atorvastatin. HepG2 cells were seeded in 96-well culture plates at a density of 1.0 × 10^4^ cells/well and cultured in DMEM medium overnight. Then, cells were treated with various concentrations of IFNγ and/or TNFα or atorvastatin for 24 and 48 h periods. When the specified period was completed, cells were incubated with MTT dye for 2 h at 37 °C until the formazan crystals were formed. Then, these crystals were dissolved in DMSO, and the resulting-colored solution was quantified using a multi-well spectrophotometer (ELISA reader). Cell viability was calculated as: % cell survival = (OD of sample/OD of control) × 100.

### 4.4. Real-Time RT-PCR

HepG2 cells at 4 × 10^5^ cells/well density were seeded in 6-well culture plates overnight and cultured in specific treatments for 6 h before being harvested for mRNA expression analysis by real-time RT-PCR. Total RNA was isolated using an illustra-RNAspin Mini RNA Isolation kit (GE Healthcare Europe GmbH, Freiburg, Germany), which was then reverse transcribed to cDNA using an iScript™ cDNA Synthesis Kit (Bio-Rad, Hercules, CA, USA). The mRNA expression from cDNA was then determined by RT-PCR using the Applied Biosystems 7500/7500 Fast Real-Time PCR system and SensiFAST™ SYBR^®^Lo-ROX (Bioline, London, UK). The cycling conditions were 95 °C for 2 min, followed by 45 cycles of 95 °C for 5 s, 60 °C for 10 s, and 72 °C for 30 s, with a final extension at 72 °C for 10 min. The expression of each gene was normalized to that of the control gene, GAPDH, as an internal control. The relative expression level (fold-changes) of the target gene was calculated by normalizing with the corresponding control. The following primer sequences were used in these assays.
human PD-L1 forward primer5′ AAATGGAACCTGGCGAAAGC-3′human PD-L1 reverse primer5′ GATGAGCCCCTCAGGCATTT-3′human GAPDH forward primer5′TGGTATCGTGGAAGGACTCATGAC-3′human GAPDH reverse primer 5′ATGCCACTCAGCTTCCCGTTCAGC-3′

### 4.5. Western Blotting Assay

HepG2 cells were seeded in 6-well culture plates at 4 × 10^5^ cells/well density overnight and cultured in specific treatments for 24 h before being harvested for analysis by western blot assay. Cells were lysed using radioimmunoprecipitation assay (RIPA) buffer, and the supernatant obtained was subjected to electrophoresis using 12% SDS-polyacrylamide to separate proteins. Then, the proteins were transferred to nitrocellulose membranes (GE Healthcare Europe GmbH, Freiburg, Germany) and probed with specific primary antibodies after being blocked with 5% skim milk in phosphate-buffered saline with Tween 20 (PBS-T) for 1 hour at room temperature. Membranes were incubated with a primary antibody (1:1000 dilution) overnight at 4 °C, which were then rinsed off three times and incubated again with an appropriate secondary antibody (1:1000 dilution) for 1 h at room temperature. The protein bands on the membranes were detected using the ChemiDoc XRS system (Bio-Rad, Hercules, CA, USA) after being exposed to an ECL substrate enhancer (SuperSignal West Femto Substrate, Thermo Scientific). The band density was then analyzed using TotalLab TL120 software. The level of β-actin was served as a loading control. After one target protein was detected, the primary and secondary antibody complex was stripped off using the stripping buffer (Thermo Scientific) and, the membrane was re-probed with another primary antibody and detected again in the same procedure, in case of necessity.

### 4.6. Immunocytochemistry

HepG2 cells were seeded on sterile glass coverslips inside 24-well culture plates at 6 × 10^4^ cells/well density overnight and treated with specific treatments for 24 h. Cells were fixed with 4% paraformaldehyde and washed with phosphate-buffered saline with Tween 20 (PBS-T) before being blocked with 1% BSA containing glycine prepared in PBS for 1 hour at room temperature. Then, cells were incubated with specific primary antibodies that were prepared in 1% BSA (1:200 dilution for PD-L1 antibody) for overnight at 4 °C. After being washed with PBS-T three times, cells were incubated with fluorochrome-conjugated secondary antibody, anti-rabbit IgG-FITC (Alexa Fluor 488), in 1:1000 dilution for 1 h at room temperature in the dark. Stained coverslips were mounted on the glass slides with a drop of mounting media containing DAPI: ProLongTM Gold antifade reagent with DAPI (Invitrogen, Thermo Fisher Scientific). Slides were photographed under a fluorescence microscope (Scope A1, Carl Zeiss). Images were analyzed using ImageJ software.

### 4.7. Statistical Analysis

Statistical analysis was performed using SPSS software, including one-way ANOVA followed by Bonferroni’s post hoc test. Data from three independent experiments were expressed as mean ± SD or mean ± SE, and *p* values < 0.05 were considered as statistically significant.

## Data Availability

Not applicable.

## References

[1] Sung H., Ferlay J., Siegel R.L., Laversanne M., Soerjomataram I., Jemal A., Bray F. (2021). Global Cancer Statistics 2020: GLOBOCAN Estimates of Incidence and Mortality Worldwide for 36 Cancers in 185 Countries. CA Cancer J. Clin..

[2] Johnston M.P., Khakoo S.I. (2019). Immunotherapy for hepatocellular carcinoma: Current and future. World J. Gastroenterol..

[3] Chen H.J., Hu M., Xu F.G., Xu H.J., She J.J., Xia H.P. (2018). Understanding the inflammation-cancer transformation in the development of primary liver cancer. Hepatoma. Res..

[4] Fu J., Xu D., Liu Z., Shi M., Zhao P., Fu B., Zhang Z., Yang H., Zhang H., Zhou C. (2007). Increased regulatory T cells correlate with CD8 T-cell impairment and poor survival in hepatocellular carcinoma patients. Gastroenterology.

[5] Arasanz H., Gato-Canas M., Zuazo M., Ibanez-Vea M., Breckpot K., Kochan G., Escors D. (2017). PD1 signal transduction pathways in T cells. Oncotarget.

[6] Kythreotou A., Siddique A., Mauri F.A., Bower M., Pinato D.J. (2018). Pd-L1. J. Clin. Pathol..

[7] Solinas A., Calvisi D.F. (2016). Programmed death ligand 1 expression in hepatocellular carcinoma: A prognostic marker and therapeutic target for liver cancer?. Hepatology.

[8] Abiko K., Matsumura N., Hamanishi J., Horikawa N., Murakami R., Yamaguchi K., Yoshioka Y., Baba T., Konishi I., Mandai M. (2015). IFN-gamma from lymphocytes induces PD-L1 expression and promotes progression of ovarian cancer. Br. J. Cancer.

[9] Xie Q.K., Zhao Y.J., Pan T., Lyu N., Mu L.W., Li S.L., Shi M.D., Zhang Z.F., Zhou P.H., Zhao M. (2016). Programmed death ligand 1 as an indicator of pre-existing adaptive immune responses in human hepatocellular carcinoma. Oncoimmunology.

[10] Schoop R., Wahl P., Le Hir M., Heemann U., Wang M., Wuthrich R.P. (2004). Suppressed T-cell activation by IFN-gamma-induced expression of PD-L1 on renal tubular epithelial cells. Nephrol. Dial. Transpl..

[11] Lim S.O., Li C.W., Xia W., Cha J.H., Chan L.C., Wu Y., Chang S.S., Lin W.C., Hsu J.M., Hsu Y.H. (2016). Deubiquitination and Stabilization of PD-L1 by CSN5. Cancer Cell.

[12] Hartley G., Regan D., Guth A., Dow S. (2017). Regulation of PD-L1 expression on murine tumor-associated monocytes and macrophages by locally produced TNF-alpha. Cancer Immunol. Immunother..

[13] Li N., Wang J., Zhang N., Zhuang M., Zong Z., Zou J., Li G., Wang X., Zhou H., Zhang L. (2018). Cross-talk between TNF-alpha and IFN-gamma signaling in induction of B7-H1 expression in hepatocellular carcinoma cells. Cancer Immunol. Immunother..

[14] Stancu C., Sima A. (2001). Statins: Mechanism of action and effects. J. Cell Mol. Med..

[15] Yi C., Song Z., Wan M., Chen Y., Cheng X. (2017). Statins intake and risk of liver cancer: A dose-response meta analysis of prospective cohort studies. Medicine.

[16] Sarrabayrouse G., Pich C., Teiti I., Tilkin-Mariame A.F. (2017). Regulatory properties of statins and rho gtpases prenylation inhibitiors to stimulate melanoma immunogenicity and promote anti-melanoma immune response. Int. J. Cancer.

[17] Greenwood J., Steinman L., Zamvil S.S. (2006). Statin therapy and autoimmune disease: From protein prenylation to immunomodulation. Nat. Rev. Immunol..

[18] Feng X., Han D., Kilaru B.K., Franek B.S., Niewold T.B., Reder A.T. (2012). Inhibition of interferon-beta responses in multiple sclerosis immune cells associated with high-dose statins. Arch. Neurol..

[19] Chung H.K., Lee I.K., Kang H., Suh J.M., Kim H., Park K.C., Kim D.W., Kim Y.K., Ro H.K., Shong M. (2002). Statin inhibits interferon-gamma-induced expression of intercellular adhesion molecule-1 (ICAM-1) in vascular endothelial and smooth muscle cells. Exp. Mol. Med..

[20] Pardoll D.M. (2012). The blockade of immune checkpoints in cancer immunotherapy. Nat. Rev. Cancer.

[21] Jiang X., Wang J., Deng X., Xiong F., Ge J., Xiang B., Wu X., Ma J., Zhou M., Li X. (2019). Role of the tumor microenvironment in PD-L1/PD-1-mediated tumor immune escape. Mol. Cancer.

[22] Sun J., Jiang W., Tian D., Guo Q., Shen Z. (2018). Icotinib inhibits the proliferation of hepatocellular carcinoma cells in vitro and in vivo dependently on EGFR activation and PDL1 expression. Onco Targets Ther..

[23] Sabatier R., Finetti P., Mamessier E., Adelaide J., Chaffanet M., Ali H.R., Viens P., Caldas C., Birnbaum D., Bertucci F. (2015). Prognostic and predictive value of PDL1 expression in breast cancer. Oncotarget.

[24] Liu J., Liu Y., Meng L., Liu K., Ji B. (2017). Targeting the PD-L1/DNMT1 axis in acquired resistance to sorafenib in human hepatocellular carcinoma. Oncol. Rep..

[25] Darnell J.E., Kerr I.M., Stark G.R. (1994). Jak-STAT pathways and transcriptional activation in response to IFNs and other extracellular signaling proteins. Science.

[26] Gough D.J., Levy D.E., Johnstone R.W., Clarke C.J. (2008). IFNgamma signaling-does it mean JAK-STAT?. Cytokine Growth Factor Rev..

[27] Concha-Benavente F., Srivastava R.M., Trivedi S., Lei Y., Chandran U., Seethala R.R., Freeman G.J., Ferris R.L. (2016). Identification of the Cell-Intrinsic and -Extrinsic Pathways Downstream of EGFR and IFNgamma That Induce PD-L1 Expression in Head and Neck Cancer. Cancer Res..

[28] Mimura K., Teh J.L., Okayama H., Shiraishi K., Kua L.F., Koh V., Smoot D.T., Ashktorab H., Oike T., Suzuki Y. (2018). PD-L1 expression is mainly regulated by interferon gamma associated with JAK-STAT pathway in gastric cancer. Cancer Sci..

[29] Gong A.Y., Zhou R., Hu G., Li X., Splinter P.L., O’Hara S.P., LaRusso N.F., Soukup G.A., Dong H., Chen X.M. (2009). MicroRNA-513 regulates B7-H1 translation and is involved in IFN-gamma-induced B7-H1 expression in cholangiocytes. J. Immunol..

[30] Zhang X., Zeng Y., Qu Q., Zhu J., Liu Z., Ning W., Zeng H., Zhang N., Du W., Chen C. (2017). PD-L1 induced by IFN-gamma from tumor-associated macrophages via the JAK/STAT3 and PI3K/AKT signaling pathways promoted progression of lung cancer. Int. J. Clin. Oncol..

[31] Lee S.K., Seo S.H., Kim B.S., Kim C.D., Lee J.H., Kang J.S., Maeng P.J., Lim J.S. (2005). IFN-gamma regulates the expression of B7-H1 in dermal fibroblast cells. J. Dermatol. Sci..

[32] Liu J., Hamrouni A., Wolowiec D., Coiteux V., Kuliczkowski K., Hetuin D., Saudemont A., Quesnel B. (2007). Plasma cells from multiple myeloma patients express B7-H1 (PD-L1) and increase expression after stimulation with IFN-{gamma} and TLR ligands via a MyD88-, TRAF6-, and MEK-dependent pathway. Blood.

[33] Kondo A., Yamashita T., Tamura H., Zhao W., Tsuji T., Shimizu M., Shinya E., Takahashi H., Tamada K., Chen L. (2010). Interferon-gamma and tumor necrosis factor-alpha induce an immunoinhibitory molecule, B7-H1, via nuclear factor-kappaB activation in blasts in myelodysplastic syndromes. Blood.

[34] Guerra B., Recio C., Aranda-Tavio H., Guerra-Rodriguez M., Garcia-Castellano J.M., Fernandez-Perez L. (2021). The Mevalonate Pathway, a Metabolic Target in Cancer Therapy. Front. Oncol..

[35] Omori M., Okuma Y., Hakozaki T., Hosomi Y. (2019). Statins improve survival in patients previously treated with nivolumab for advanced non-small cell lung cancer: An observational study. Mol. Clin. Oncol..

[36] Okoye I., Namdar A., Xu L., Crux N., Elahi S. (2017). Atorvastatin downregulates co-inhibitory receptor expression by targeting Ras-activated mTOR signalling. Oncotarget.

[37] Lee S.J., Qin H., Benveniste E.N. (2007). Simvastatin inhibits IFN-gamma-induced CD40 gene expression by suppressing STAT-1alpha. J. Leukoc. Biol..

[38] Ma X., Bi E., Lu Y., Su P., Huang C., Liu L., Wang Q., Yang M., Kalady M.F., Qian J. (2019). Cholesterol Induces CD8(+) T Cell Exhaustion in the Tumor Microenvironment. Cell Metab..

[39] Cerezo M., Guemiri R., Druillennec S., Girault I., Malka-Mahieu H., Shen S., Allard D., Martineau S., Welsch C., Agoussi S. (2018). Translational control of tumor immune escape via the eIF4F-STAT1-PD-L1 axis in melanoma. Nat. Med..

[40] Nowicki T.S., Hu-Lieskovan S., Ribas A. (2018). Mechanisms of Resistance to PD-1 and PD-L1 Blockade. Cancer J..

